# A CNN Model for Physical Activity Recognition and Energy Expenditure Estimation from an Eyeglass-Mounted Wearable Sensor

**DOI:** 10.3390/s24103046

**Published:** 2024-05-11

**Authors:** Md Billal Hossain, Samuel R. LaMunion, Scott E. Crouter, Edward L. Melanson, Edward Sazonov

**Affiliations:** 1 Department of Electrical and Computer Engineering, The University of Alabama, Tuscaloosa, AL 35487, USA; mhossain14@crimson.ua.edu; 2 Department of Kinesiology, Recreation and Sport Studies, The University of Tennessee, Knoxville, TN 37996, USA; samuel.lamunion@nih.gov (S.R.L.); scrouter@utk.edu (S.E.C.); 3 USA Division of Endocrinology, Metabolism, and Diabetes, University of Colorado Anschutz Medical Campus, Aurora, CO 80045, USA; ed.melanson@cuanschutz.edu

**Keywords:** physical activity recognition, wearable sensor device, energy expenditure, energy intake

## Abstract

Metabolic syndrome poses a significant health challenge worldwide, prompting the need for comprehensive strategies integrating physical activity monitoring and energy expenditure. Wearable sensor devices have been used both for energy intake and energy expenditure (EE) estimation. Traditionally, sensors are attached to the hip or wrist. The primary aim of this research is to investigate the use of an eyeglass-mounted wearable energy intake sensor (Automatic Ingestion Monitor v2, AIM-2) for simultaneous recognition of physical activity (PAR) and estimation of steady-state EE as compared to a traditional hip-worn device. Study data were collected from six participants performing six structured activities, with the reference EE measured using indirect calorimetry (COSMED K5) and reported as metabolic equivalents of tasks (METs). Next, a novel deep convolutional neural network-based multitasking model (Multitasking-CNN) was developed for PAR and EE estimation. The Multitasking-CNN was trained with a two-step progressive training approach for higher accuracy, where in the first step the model for PAR was trained, and in the second step the model was fine-tuned for EE estimation. Finally, the performance of Multitasking-CNN on AIM-2 attached to eyeglasses was compared to the ActiGraph GT9X (AG) attached to the right hip. On the AIM-2 data, Multitasking-CNN achieved a maximum of 95% testing accuracy of PAR, a minimum of 0.59 METs mean square error (MSE), and 11% mean absolute percentage error (MAPE) in EE estimation. Conversely, on AG data, the Multitasking-CNN model achieved a maximum of 82% testing accuracy in PAR, a minimum of 0.73 METs MSE, and 13% MAPE in EE estimation. These results suggest the feasibility of using an eyeglass-mounted sensor for both PAR and EE estimation.

## 1. Introduction

Energy intake in humans comes from consuming food and beverages, while energy expenditure (EE) results from various physiological processes and physical activities. Specifically, EE comprises the resting metabolic rate, thermic effect of feeding, physical activity-induced energy, and non-exercise activity thermogenesis. The thermic effect of feeding accounts for a small fraction of EE, typically 8–15% [1]. The most variable forms of EE are physical activity-induced energy and non-exercise activity thermogenesis, which can be controlled through physical activity and exercise such as walking or running [2]. EE is approximately constant in a steady state of a specific physical activity (e.g., walking at a constant pace). However, EE is not constant during physical activity transitions (e.g., start running after walking). The intensity of EE is expressed in kilojoules (kJ) or kilocalories (kcal) per unit of time and body weight, or as metabolic equivalents (METs). EE in humans is typically determined using indirect calorimetry (e.g., metabolic carts, portable metabolic systems, or the doubly labeled water method). Although those methods provide higher accuracy, they are not practical for application in the general population. Therefore, researchers are exploring different technological approaches to estimate EE in daily life.

Energy balance refers to the state where the energy intake of an individual is equivalent to their EE [3]. Imbalances in energy intake and EE lead to alterations in body mass [4]. Sustained positive energy balance (i.e., energy intake > energy expenditure) results in weight gain, with approximately 60–80% of the gained weight attributed to body fat [5,6]. Conversely, sustained negative energy balance (energy intake < energy expenditure) results in a loss in body mass. Positive energy balance, often associated with overeating and a sedentary lifestyle, has become a significant concern in modern society due to the potential risk of metabolic syndromes such as type-2 diabetes, obesity, hypertension, dyslipidemia, stroke, and cardiovascular disease. The possible solution to minimize the positive imbalance is to reduce overeating and increase physical activity. Increasing physical activity increases EE, which minimizes the effect of positive imbalance and helps control weight gain [7,8,9]. The prevalence of metabolic syndromes has significantly increased, highlighting the need for a comprehensive energy intake, EE, and physical activity monitoring system.

Physical activity refers to the movement of skeletal muscles that results in EE [10]. Physical Activity Recognition (PAR) aims to identify the type of physical activity, ranging from simple to complex activities. Once identified, the EE of a given activity can be estimated using standard values or individually calibrated values [11]. PAR is an actively researched topic due to its various real-world applications in areas such as human-computer interaction [12,13], security and surveillance systems [14,15], and healthcare systems [16,17,18,19]. In some studies [20,21], researchers have integrated PAR and EE estimation through wearable technology.

Wearable sensor devices are becoming increasingly popular due to their portability, light weight, and application diversity. Researchers are experimenting with different sensors, such as accelerometers, gyroscopes, heart rate monitors, and temperature sensors, in different body locations, such as the waist, chest, arm, leg, hip, and head. Among sensors, the accelerometer-gyroscope combination (Inertial Measurement Unit (IMU)) is frequently used due to its ability to measure multiple aspects of human motion [22,23]. The hip and wrist are the most popular body positions for the IMU-based PAR and EE estimations. Research has shown that data collected from wrist-worn devices (for example, the ActiGraph GT3X+ [24]) can overestimate EE compared to hip-worn devices [25]. A large-scale experiment [26] tested PAR and EE estimation performance using six different wear positions (wrist, hip, ankle, upper arm, and thigh). It concluded that the hip position was the best for both PAR and EE estimation. Other experiments have introduced wearables (eSense) and head-worn sensors placed in headbands for PAR [27,28]. To the best of our knowledge, no research has been conducted on simultaneously estimating PAR and EE using eyeglass-mounted head-worn wearable sensor devices. Eyeglass-mounted, head-worn wearable sensors may offer new possibilities. Location on the frame of eyeglasses may allow to integrate PAR and EE monitoring with energy intake monitoring through a variety of eating detection methods [29,30]. Traditional wearable devices for PAR and EE estimation are incapable of tracking energy intake, which presents a significant advantage for eyeglass-mounted wearable sensor devices that naturally follow the eye gaze during eating and could be used to capture images of food. Eyeglasses are a prevalent wearable, with over 75% of American [31] and 64% of global [32] adults wearing glasses for vision correction. The use of PAR sensors by regular eyeglass users may potentially reduce the user burden and improve compliance with wear. Since the movement patterns of the head are different from those at the hip or wrist, this paper attempts to evaluate the accuracy of PAR and EE estimations with respect to established methodology. Additionally, it also features hands-free operation and passive monitoring, making it the ideal choice for our research experiment. While there are minor considerations, such as the added weight, these benefits make them a promising option.

## 2. Previous Studies and Our Contribution

Traditionally, PAR and EE estimation models were developed based on classical signal processing techniques [21,23,26], statistical and classical machine learning [20]. Nevertheless, the superior performance of deep learning, such as convolutional neural networks (CNNs), long short-term memory (LSTM), and attention mechanisms, makes them attractive for PAR and EE estimation applications [33,34]. In [35], CNN and LSTM with self-attention mechanisms improved PAR performance, and a similar experiment in [36] enhanced arm exercise activity recognition. CNN-LSTM models showed improved performance and were used by other recent researchers [37,38]. The real-time feasibility of the IMU sensor and CNN-based PAR was introduced in [39]. A soft-voting and self-learning-based PAR method was proposed in [40], where the researcher enhanced accuracy by employing multiple machine learning models. The researcher also utilized a self-training mechanism to generate training data and retrain the model iteratively. Furthermore, a study in [41] proposed CNN-based EE estimation for ambulatory activities, reporting a lower error with 30 participants and five activities. In [42], multilayer perceptron (MLP) was applied to EE estimation data from 102 healthy adults engaged in two structured activities. A graph-based PAR method is proposed in [43], incorporating a combination of the deterministic selection algorithm, the lion optimization algorithm, and the association pattern algorithm (referred to as gSpan).

Notably, two significant research experiments involve multitasking models for PAR and EE estimation. Multitasking is superior to the single-task model when applied to a collection of related tasks using shared representations to learn [44]. These shared representations increase data efficiency and potentially yield faster learning and inference speeds. Additionally, simultaneously monitoring physical activity and estimating EE offers several advantages, including a more comprehensive understanding of an individual’s energy balance and activity levels, allowing for real-time tracking of EE during different activities, aiding in personalized activity recommendations, and enhancing the accuracy of energy balance assessments. Moreover, it can provide valuable insights into the relationship between activity patterns and energy intake, facilitating more effective strategies for weight management and overall health improvement [45]. In [46], a multitasking LSTM network classified activity types and estimated activity intensity using raw sensor data and MET values. Another multitasking model for PAR and EE estimation was proposed in [47], based on computer vision and heart rate sensors. Although substantial progress has been achieved, a significant research scope remains unexplored in multitasking model development for PAR and EE estimation.

The primary objective of this pilot study is to investigate the viability of using the head-mounted wearable device known as the ‘Automatic Ingestion Monitor version 2 (AIM-2) [48]’ for comprehensive estimation of PAR and steady-state EE during six different physical activities, utilizing a multitasking model. Our contributions are as follows:

- For the first time, we demonstrated the feasibility of using an eyeglass-based wearable sensor (AIM-2) for simultaneous PAR and EE estimation.
- We proposed a multitasking CNN (Multitasking-CNN) model for comprehensive estimation of PAR and EE. We employ a two-step progressive training approach to enhance the model’s performance.
- We compared the performance of Multitasking-CNN in PAR and EE estimation using data from both the hip-worn ActiGraph GT9X device and the head-mounted AIM-2 device.

## 3. Materials and Methods

### 3.1. Wearable Sensor Devices

**Figure 1:**
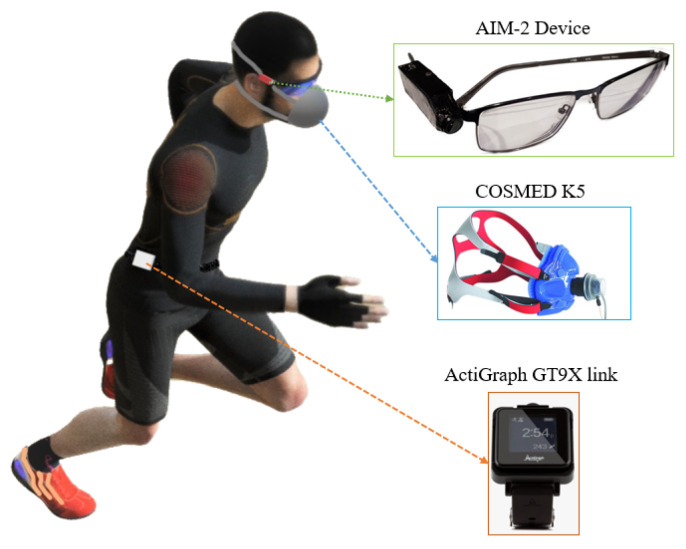
Data collection by wearable sensor devices for the Multitasking-CNN model.

Two wearable sensor devices collected the experimental data: AIM-2 and ActiGraph GT9X (AG) [49]. AIM-2 is an eyeglass-mounted passive wearable device primarily designed for dietary assessment (Figure 1). AIM-2 consists of a miniature 5 Megapixel camera with a 170-degree wide-angle gaze-aligned lens, a low-power 3D accelerometer (ADXL362 from Analog Devices, Norwood, MA, USA), and a gyroscope (LSM6DS3TR-C from STMicroelectronics N.V.). The IMU from AIM-2 has a full-scale range of ±8 g and ±2000 degrees per second, respectively. Data from the AIM-2 IMU were sampled at 128 Hz and stored on an SD card. The AIM-2 can be attached to any eyeglass using simple, double-sided adhesive, eliminating the need for a specialized eyeglass frame.

AG is a lightweight (14 g) and non-invasive commercially available wearable sensor device that is widely used in the PAR research community [33]. The AG device is equipped with a triaxial primary accelerometer (full scale range ± 8 g), a triaxial gyroscope (full scale range ± 2000 degrees per second), a triaxial magnetometer (full scale range ± 4800 micro-Tesla), and an internal sensor thermometer to measure the temperature inside the sensor housing. The primary accelerometer in the AG device has a selectable sampling rate of 30–100 Hz (90 Hz selected), and the gyroscope has a fixed sampling rate of 100 Hz.

A portable indirect calorimeter (COSMED K5) [50] was used for collecting the ground truth EE. The COSMED K5 measures oxygen consumption and carbon dioxide production using a breath-by-breath or mixing chamber mode. The COSMED K5 unit was calibrated prior to each use using a five-step manufacturer specified procedure, including room air calibration, carbon dioxide scrubber calibration, reference gas calibration using a mixture of 16.0% O_2_, 5.0% CO_2_, and balanced nitrogen, volume calibration using a 3-liter Hans Rudolf syringe, and delay calibration to check the timing of air flow from the face mask to the analyzers. The accuracy of the device is reported in [51].

### 3.2. Study Design

**Figure 2:**
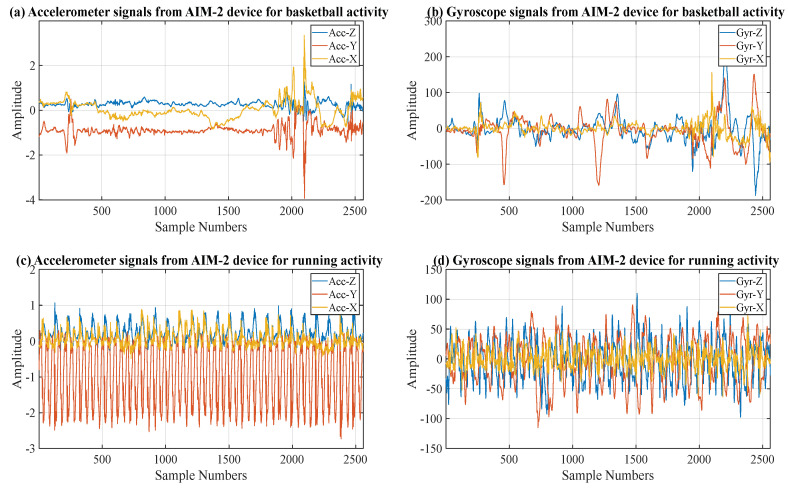
Sample accelerometer and gyroscope raw sensor data (20 s) collected from participant 101 by the AIM-2 device ((**a**,**b**): basketball activity; (**c**,**d**): running activity).

A study was conducted at The University of Tennessee, Knoxville, with six participants (mean ± SD; age 23.8 ± 3.9 y, BMI 24.25 ± 5.77 kg/m^2^, body weight 77.1 ± 20.2 kg; 5 males, 1 female). The University of Tennessee Knoxville Institutional Review Board approved the study protocol (IRB#: UTK IRB-14-01988-XP). Participants’ height and weight were measured at the beginning of the study visit. All measurements were taken without shoes and in lightweight clothing. Height was measured using a wall-mounted stadiometer, and weight was taken using a calibrated physician’s scale. Participants completed the following six structured activities for six minutes per activity during a single laboratory visit: seated computer work, sweeping, stationary cycling (50 W), variable treadmill walking (3 mph, 0% grade; 4 mph, 0% grade; 3 mph, 5% grade), treadmill running (6 mph, 0% grade), and one-vs.-one basketball. The AG was worn on the right hip, an AIM-2 was attached to the right arm of a pair of non-prescription eyeglasses, and a COSMED K5 was worn to measure gas exchange throughout the activity protocol (Figure 1). A human annotator annotated each activity’s start and stop times to obtain the periods of interest. Table 1 shows the summary of the duration of each activity (six participants identified by participant ID 101–106). The computer activity was discarded for participant 105 because the AG device started recording data after starting the computer activity data collection. Figure 2 displays the raw sensor signal samples collected by the AIM-2 device from participant 101 across various activities.

After each study session, breath-by-breath (averaged into a 30-s window) EE data were extracted from the COSMED OMNIA software. The relative VO_2_ (mLkg^−1^min^−1^) was obtained by dividing the mean VO_2_ (mLmin^−1^) from each 30-second epoch by the participant’s body mass in kg. For all weight-bearing activities, an additional 2 kg of weight was added to the body weight (the weight of the K5 unit and harness system). The EE in METs was then obtained by dividing the relative VO_2_ (mLkg^−1^min^−1^) by 3.5 mLkg^−1^min^−1^. The last 30-s window was discarded to obtain steady-state EE, and the average of the four preceding 30 s windows of data was used as the steady-state EE for each activity. The steady-state EE value for each activity was then matched by timestamp and criterion activity label to the corresponding 10 s activity periods for each participant. The transition between the activities was not considered for analysis. All analyses in this paper refer to steady-state EE estimation.

### 3.3. Study Data Processing

#### 3.3.1. IMU Signal Analysis

Due to historical reasons, the AG device sampled acceleration at 90 Hz and the gyroscope at 100 Hz [52]. The accelerometer signal was up-sampled to 100 Hz using the MATLAB resample function to ensure the uniformity of the signals. The gyroscope signals from the AIM-2 and AG devices were clipped to ±400 degrees per second to remove extreme outliers. We did not apply any filters to remove the noise from the IMU signal. The accelerometer and clipped gyroscope sensor signals were used in the next step for sensor signal-to-image conversion.

#### 3.3.2. Sensor Signal to Digital Image Conversion

In this step, sensor signals were represented as grayscale images to facilitate the application of convolutional neural networks that compute features used in PAR and EE estimation. First, each channel (X, Y, and Z) of the accelerometer and gyroscope sensor signals was rescaled individually to [0,255] by Equation (1).

(1) $$X_{scaled}=NM+\left[\frac{X-X_{min}}{X_{max}-X_{min}}\right]\times NMa$$

where *NM* = 0 is the new minima; *NMa* = 255 is the new maxima; *X_scaled_* = scaled sensor signal; *X* = sensor signal; *X_min_* = minimum value of sensor signal; and *X_max_* = maximum value of sensor signal. The values of $X_{min}$ and $X_{max}$ were obtained with the bounds function in MATLAB.

**Figure 3:**
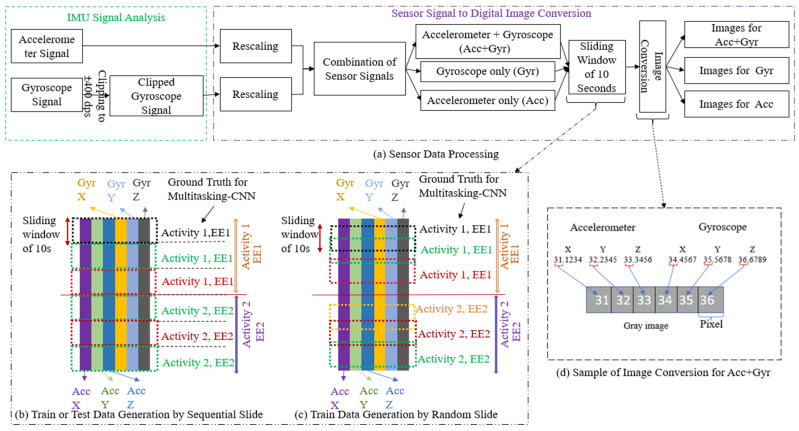
Sensor data processing for Multitasking-CNN model development.

Then, the fractional part of the converted value was removed with floor operation (Figure 3d) in MATLAB. Next, the rescaled sensor signals were grouped together into the following three combinations: accelerometer and gyroscope (Acc+Gyr); accelerometer only (Acc); and gyroscope only (Gyr), as shown in Figure 3.

**Figure 4:**
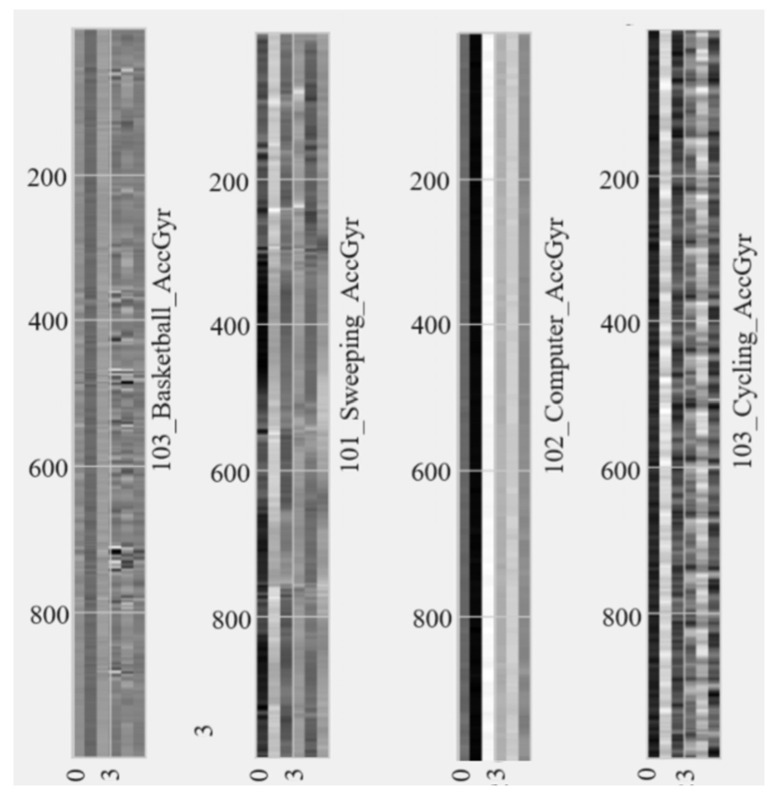
Sample final output (grayscale image) of sensor signal to image conversion for different participants and activities for Acc+Gyr.

A sliding window of 10 s was applied to the grouped sensor data, which was then saved as a grayscale image (Figure 4). The choice of window size significantly affects EE and PAR performance. A shorter window aids in fast-tracking and transitions but may result in false positives. Previous studies [53] suggest a 10 s window with a 55 Hz sampling rate is optimal for activity recognition. Further research [54] examined various sensors and data from 20 participants in 52 activities, indicating that longer windows generally improve PAR, except for short activities. A parallel trend emerged in EE-focused research articles. For instance, in [42], researchers utilized a 10 s window for EE estimation. Subsequently, a more exhaustive study detailed in [55], encompassing 100 young participants, underscored that a 10-s window size yields a reduced estimation error for EE. Hence, we chose a 10 s window for a balanced PAR and EE estimation performance. The sliding window operation was performed for each activity and participant’s data, with no overlap between windows from different activities or participants.

The training and validation datasets were produced using sequential and random windows (Figure 3b,c). The sequential window was precisely aligned with the activity boundaries, and the ground truth was generated based on the corresponding activity label and EE (Figure 3b). In contrast, the starting point of random windows (Figure 3c) was selected randomly, which can be considered a form of data augmentation. The testing dataset was produced using only sequential windows. Grayscale images from the sensor combination Acc+Gyr, Acc, and Gyr were saved for both AIM-2 and AG devices. Image tensor sizes (heigh×twidth×chanel) were 1280 × 6 × 1 for Acc+Gyr, 1280 × 3 × 1 for Acc, 1280 × 3 × 1 for Gyr in AIM-2; and 1000 × 6 × 1 for Acc+Gyr, 1000 × 3 × 1 for Acc, 1000 × 3 × 1 for Gyr in AG.

### 3.4. Proposed Model for PAR and EE Estimation

**Figure 5:**
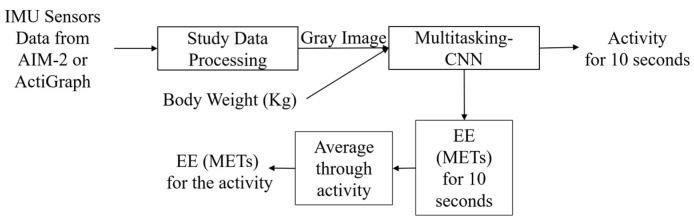
Overall architecture of PAR and EE estimations.

Our proposed model (Figure 5) integrates the computation of PAR and EE estimations into a single operation. The IMU sensor data are converted into grayscale images and supplied as input to the Multitasking-CNN model. Given the pivotal role of body weight in determining EE, this value was incorporated as an additional external input to the Multitasking-CNN. Multitasking-CNN comprises a CNN model with a fully connected dense layer. Despite the computational complexity of CNN-based deep learning models, they eliminate the need for handcrafted feature selection, a step in traditional signal processing methods. The latest microcontrollers include low-power hardware for acceleration of inference in deep neural networks, thus enabling the use of deep learning in wearables. Therefore, we opted for a deep learning-based approach. The subsequent section provides a more detailed description of Multitasking-CNN.

### 3.5. Multitasking-CNN Architecture

**Figure 6:**
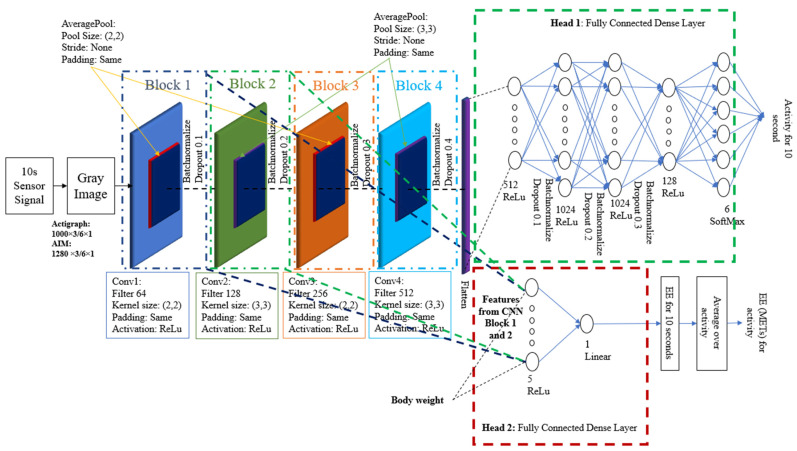
Multitasking-CNN model.

Multitasking demonstrates superiority over the single-task model when applied to a set of interrelated tasks. Therefore, we chose to adopt a multitasking architecture for our experiment. A typical multitasking model architecture is designed to handle multiple related tasks simultaneously. Generally, a multitasking model consists of an input layer, a shared layer, a task-specific layer, and a head. In Multitasking-CNN (Figure 6), the shared layer consists of the CNN blocks that apply filters to the input data by performing convolution operations. Each CNN feature extraction block consists of a series of layers, including convolution, average pooling, batch normalization, and dropout. A head is the final part of a multitasking model that produces the desired outputs or predictions. The proposed Multitasking-CNN model has a separate head for PAR and EE estimation (Head-1 and Head-2 in Figure 6).

To determine the optimal configuration for the CNN blocks (number of kernels, filter size, and the number of neurons in dense layers), a grid search technique was employed by monitoring the model’s PAR performance. Twelve combinations (as presented in Table 2 were evaluated and compared to determine the optimal CNN feature block combination for EE estimation. As an example, in Table 2, the entry ‘block 1 + 2 + 3’ signifies that Head-2 was trained using the features extracted from the output of CNN blocks 1, 2, and 3. The Multitasking-CNN model achieved the best EE estimation when Head-2 took features from CNN blocks 1 and 2. Thus, this configuration was selected as our proposed model and used for model performance analysis. The model predicts the EE over a 10-s interval, and the final steady-state EE was calculated as the average EE across the entire activity.

### 3.6. Multitasking-CNN Training

The commonly used multitasking training approaches are hard parameter sharing, soft parameter sharing, task-specific layers, progressive training, and reinforcement learning with auxiliary tasks [56]. In this study, we evaluated two-step progressive training and one-step hard parameter sharing approaches.

In the two-step progressive training, we trained the model for the task of PAR (Head-1). The model weights were then held constant, and Head-2 was trained for EE estimation.

PAR is a classification problem, whereas EE estimation is a regression problem. Thus, during training, the categorical cross-entropy loss function (Equation (2)) was used for PAR and the mean squared error loss function (Equation (3)) for EE estimation.

In the one-step hard parameter sharing approach, the model was trained for both PAR and EE estimation simultaneously, utilizing a combined loss function. The combined loss was calculated based on a weighted combination of PAR and EE estimation losses (Equation (4)). The optimal values of $w_{1}$ and $w_{2}$ were found by a grid search.

(2) $${Loss}_{PAR}=-\sum_{c=1}^{M}y_{i,c}\log\left(P_{i,c}\right)$$

where M=number of classes and i=observation number.

(3) $${Loss}_{EE}=\frac{1}{n}\sum_{i=1}^{n}{{(y}_{i}-\hat{y_{i}})}^{2}$$

where $y_{i}=actual\;value\;and\;\hat{y_{i}}=predicted\;value$.

(4) $${Loss}_{combined}={w_{1}\times Loss}_{PAR}+w_{2}\times {Loss}_{EE}\;\mathrm{and}\;w_{1}+w_{2}=1$$

The model’s performance was evaluated by a 6-fold leave-one-out procedure, where the entire data from one (different for each fold) participant was used as the test set. The data from the remaining 5 participants were split by a 3:1 ratio into the training and validation sets, respectively. The training/validation/testing procedures were repeated 6 times, so that on each of the 6 folds, the training and validation data were completely independent from the testing data. The average performance across 6 folds was measured in terms of accuracy, precision, recall, and F1 score (equations are given in [32]) for the PAR classification problem and mean squared error (MSE) (Equation (5)) and mean absolute percentage error (MAPE) (Equation (6)) for the EE estimation regression problem. The following hyperparameters were used during training: a batch size of 100, an initial learning rate of 0.00002 with a 50% decay after 10 epochs, and a maximum of 200 epochs with an early stopping criterion at a patience value of 25.

(5) $$MSE\left(y,\hat{y}\right)=\frac{1}{N}\;\sum_{i=0}^{N-1}{\left(y_{i}-\hat{y_{i}}\right)}^{2}\;$$

(6) $$MAPE=\left(\frac{1}{N}\sum_{i=0}^{P-1}\frac{\left|y_{i}-\hat{y_{i}}\right|}{y_{i}}\;\right)$$

where $N=Sample\;Number,\;y=actual,\;and\;\hat{y}=predicted\;value$.

## 4. Results and Discussion

### 4.1. Two-Step Progressive Training

The mean accuracy, precision, recall, and F1 score for both the AIM-2 and AG devices are reported in Table 3. The highest (highlighted in bold) average PAR testing accuracy of 95% was achieved by a model utilizing gyroscope data from the AIM-2 device. In comparison, a testing accuracy of 82% was attained using accelerometer and gyroscope data from the AG device. The table provides evidence that the model achieves higher PAR performance with AIM-2 collected data compared to AG collected data, regardless of sensor combination.

Table 2 displays the MSE and MAPE results for twelve different CNN block combinations using accelerometer data. The MSE and MAPE values for Multitasking-CNN with various sensor combinations are tabulated in Table 4. The findings from both Table 2 and Table 4 show the features from CNN blocks 1 and 2 offer good performance (highlighted in bold) for EE estimation. Table 4 lists the testing MSE and MAPE for EE estimation for the two-step progressive training approach. The Multitasking-CNN model achieved the lowest (highlighted with bold) MSE and MAPE values for the AIM-2 device-collected data. The MSE value was 0.59 METs, and the MAPE value was 11%. In comparison, the AG-collected data had higher values, with an MSE of 0.73 METs and a MAPE of 12%.

The model achieves the lowest MSE and MAPE for accelerometer and gyroscope sensors in combination with the AIM-2 device-collected data. For the accelerometer sensor combination, the AIM-2 data result in an MSE of 0.75 METs and a MAPE of 14%, which is highly competitive compared to the AG device data with an MSE of 0.73 METs and a MAPE of 12%. The AG device data outperform the EE estimation with the gyroscope sensor (compared to AIM-2 gyroscope data). However, when considering overall performance, the AIM-2 device achieves the lowest or, at the very least, comparable results when compared to the AG device data.

**Figure 7:**
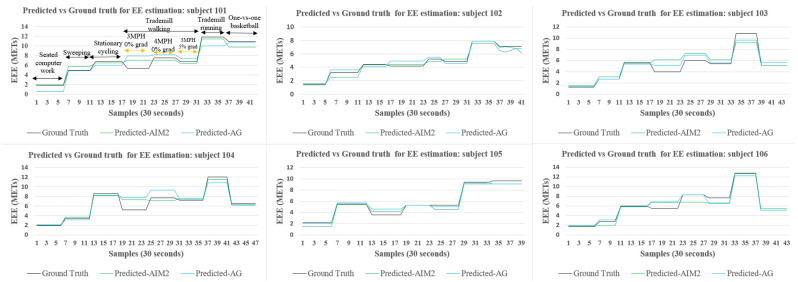
AG (Acc) vs. AIM-2 (Acc+Gyr) for EE estimation with progressive training.

Figure 7 presents the predicted vs. actual EE estimation for the best-performing models with data from the AIM-2 and AG devices. The EE estimation is depicted for six activities, including “seated computer work”, “sweeping”, “stationary cycling”, “treadmill walking with different paces”, “treadmill running”, and “one-vs.-one basketball” in that order. Note that participant 105 did not perform “seated computer work”, so “sweeping” was their first activity.

Overall, the results from two-step progressive training indicate that the AIM-2 device data yield higher PAR accuracy with testing accuracy values of 91%, 88.0%, and 95%, compared to the AG device data, which had testing accuracy values of 82%, 78.0%, and 80%. For the AIM-2 device, the combination of accelerometer and gyroscope sensors achieved the lowest MSE of 0.59 METs and MAPE of 11%, while for the AG device, the accelerometer sensor yielded the best EE estimation performance (MSE of 0.73 METs and MAPE of 12%). The Multitasking-CNN demonstrated a good prediction for “sweeping”, “stationary cycling”, “treadmill running”, and “one-vs.-one basketball” for both the AIM-2 and AG device data. Our proposed Multitasking-CNN model, trained in two steps, demonstrates superior PAR and EE predictions for both AIM-2 and AG device collected data.

### 4.2. One-Step Hard Parameter Sharing Training

Table 5 shows the results of the grid search for parameters $w_{1}$ and $w_{2}$. The results in the table show that the higher the value of $w_{1}$, the better the PAR performance is (accuracy, precision, recall, and F1 score). This is an expected behavior, as the loss function for PAR is prioritized over the EE loss function. However, the reverse effect was not observed if the value of $w_{2}$ was higher ($w_{1}$ is lower in Table 5). The MSE and MAPE were higher when $w_{2}$ was set at 0.9 (i.e., $w_{1}$ = 0.1). The results from Table 5 suggest that a combination of $w_{1}$ = 0.3 and $w_{2}$ = 0.7 provides balanced performance in both PAR and EE estimation for the accelerometer sensor. Similar results were obtained for ‘Acc+Gyr’ and ‘Gyr’ sensor combinations.

Table 6 shows the PAR performance of one-step training for different sensor combinations. The model achieved the best PAR accuracy for gyroscope data from AIM-2, having an accuracy of 88%, 91% precision, 88% recall, and an 89% F1 score. AG device data achieve a maximum of 80%, 78%, 80%, and 79% accuracy, precision, recall, and F1 score with accelerometer sensor data. The table also shows that the model achieves higher PAR performance with AIM-2 collected data compared to AG collected data, irrespective of the sensor combination.

Table 7 shows the model EE performance for AIM-2 and AG collected data by using one-step hard parameter sharing training. The model achieved the best MSE and MAPE with accelerometer sensor data for both AIM-2 and AG devices. The best-performing EE estimation model achieved minimum MSE and MAPE of 2.69 METs and 28% for AIM-2 and 2.79 METs and 30% for AG, respectively. The model achieved lower MSE and MAPE for ‘Acc’ and ‘Gyr’ sensor combination with AIM-2 collected data (with compared to AG collected data). However, when considering the ‘Acc+Gyr’ sensor combination from the AG device, the model achieved lower MSE and MAPE compared to the AIM-2 device. In summary, the AIM-2 device consistently achieved comparable results when compared to the AG device data.

The result from one-step hard parameter sharing and two-step progressive training concludes that the two-step training method achieves better performance for both PAR (maximum accuracy, precision, recall, and F1 score of 95%, 96%, 95%, and 95%, respectively) and EE estimation (lowest MSE and MAPE of 0.59 METs and 11%). The findings indicate that when utilizing eyeglass-mounted AIM-2 collected data, it consistently outperforms the AG data in terms of PAR, irrespective of the sensor combination and training strategy employed. Regarding EE estimation, the model achieved the lowest MSE and MAPE when using AIM-2 data, regardless of whether the one-step or two-step training method was employed. This research outcome suggests that eyeglass-mounted wearable sensor devices could be a potential platform for comprehensive monitoring of physical activity and energy expenditure.

Although the models achieve the highest PAR and EE estimation using different sensor combinations, there are notable variations. For instance, in the case of AIM-2 collected data, the gyroscope signal demonstrates the highest PAR accuracy (Table 3), while the accelerometer and gyroscope signals achieve low MSE and MAPE (Table 4). However, a well-balanced performance in PAR and EE estimation is consistently observed when using accelerometer and gyroscope signals, regardless of the device and training strategy employed.

Table 8 presents a comparative view of PAR and EE estimation using wearable sensor devices in various body locations. Our focus is on simultaneous PAR and EE estimation, aligning with our experiment. A direct comparison of model performance is challenging due to differences in data collection procedures, performance metrics, and physical activities. We provide a comparative overview of our experiment’s pros and cons (compared to our experiment). Our primary contribution is experimenting with simultaneous PAR and EE monitoring using the eyeglass-mounted AIM-2 device, offering new possibilities. The AIM-2 device, which can be easily affixed to any eyeglass, eliminates the need for additional gadgets, a significant advantage over other methods (Table 8). Our AIM-2-based experiments also offer advantages such as collecting ground truth EE from COSMED-K5 (compared with [45]), convenience (compared with all methods in Table 8), and no handcrafted feature selection (compared with [26]). Our experiment’s limitations are discussed in Section 5. Numerically, using the AIM-2 device, we achieved a maximum testing accuracy of 95% for PAR, a minimum MSE of 0.59 METs, and an 11% MAPE in EE estimation compared to methods listed in Table 8.

## 5. Conclusions

We have developed a multitasking model called Multitasking-CNN for estimating PAR and steady-state EE using data collected from the AIM-2 and AG devices. The model was trained using both a two-step progressive training approach and a one-step hard parameter sharing approach. Our results indicate that the two-step progressive training approach outperforms the one-step hard parameter-sharing approach. With the AIM-2 device data and two-step progressive training, the model achieves a maximum testing accuracy of 95% for PAR estimation, a minimum MSE of 0.59 METs for EE estimation, and an MAPE of 11%. Conversely, for the AG device data, the model achieves a maximum testing accuracy of 82% for PAR estimation, a minimum MSE of 0.73 METs for EE estimation, and an MAPE of 13%.

Regardless of the training strategy and sensor combination, the models consistently demonstrate higher PAR performance when using the AIM-2 data. For EE estimation, the model achieves the lowest MSE and MAPE for AIM-2 collected data, irrespective of training strategy. These findings suggest that eyeglass-mounted wearable devices hold promising potential for further research in PAR and EE estimation.

This experiment has some limitations. The human study was cut short in 2020 due to the COVID-19 pandemic, and for logistical reasons, the study could not be restarted in 2022, when human metabolic research became possible again. The small number of study participants is a major limitation that impacts the ability to draw broad conclusions. The findings may not accurately reflect the responses of broader demographic groups, such as different age ranges, ethnicities, or socioeconomic backgrounds. Moreover, biological and physiological differences between males and females could influence the outcomes of the study. With only one female participant, the result might skew toward the male gender. Finally, the narrow range of ages (from approximately 20 to 30 years) and BMI values may restrict the generalizability of the findings to other age groups or individuals with different BMI ranges. However, we used rigorous (e.g., leave-one-out) training and validation methods to derive generalizable results (based on available data) that justify further work with head-worn sensors. Future research with larger and more diverse samples is necessary to confirm and extend these results to broader populations.

Another limitation is that the experiment was carried out in a controlled environment with a small number of activities. Moreover, the experiment only compares the eyeglass-mounted AIM-2 with a hip-worn wearable sensor device. Nonetheless, our goal is to demonstrate the feasibility of using eyeglass-mounted wearable sensor devices for PAR and EE estimation. A more rigorous study with a larger population, a protocol inclusive of more activities of daily living, and more sophisticated learning models (such as LSTM or CNN-LSTM) needs to be conducted to generalize the performance. The limitations of this work will be addressed in our future work.

Future research could delve into AIM-2’s potential for comprehensive monitoring of both energy intake and EE, which would aid in achieving energy balance and potentially mitigating the risk of chronic metabolic syndromes.

## Figures and Tables

**Table 1:** Duration of data collection (in minutes) for PAR and EE estimation.

Activity		Participant ID					
		101	102	103	104	105	106
Computer		6	5.5	6	6	--	6
Sweeping		5.5	6	5.5	6	6	5
Cycling		6	5.5	6	6	6	6
Walking	3 mph (0% grade)	5.5	6.5	6	6	6	6
	4 mph (0% grade)	5.5	4.5	5.5	6	5.5	5.5
	3 mph (5% grade)	5	5.5	5.5	6	5.5	5.5
Running at 6 mph		5.5	5.5	5.5	5.5	5.5	5.5
Basketball		6	5.5	6	6	6	6
Total duration (in minutes)		45	44.5	46	47.5	40.5	45.5

**Table 2:** Testing MSE and MAPE for EE estimation using different CNN block combinations (accelerometer sensor only).

CNN Blocks to Head 2	AIM-2 Sensor Data		AG Sensor Data	
	MSE	MAPE	MSE	MAPE
Block-1	0.82	0.14	0.87	0.12
Block-2	1.75	0.18	1.40	0.19
Block-3	0.97	0.23	1.58	0.22
Block-4	2.78	0.25	3.58	0.32
Block 1 + 4	0.67	0.15	1.03	0.16
**Block 1 + 2**	**0.75**	**0.14**	**0.73**	**0.12**
**Block 1 + 3**	**0.65**	**0.13**	**0.99**	**0.15**
Block 2 + 3	1.33	0.20	1.98	0.23
Block 2 + 4	1.78	0.21	1.69	0.22
Block 1 + 2 + 3	0.70	0.14	0.91	0.13
Block 2 + 3 + 4	1.34	0.20	1.70	0.23
Block 1 + 2 + 3 + 4	0.77	0.15	0.88	0.15

**Table 3:** Average testing accuracy, precision, recall, and F1 score (testing) for par with two-step progressive training.

Average Metric (%)	Using AIM-2 Sensor Data			Using AG Sensor Data		
	Acc+Gyr	Acc	Gyr	Acc+Gyr	Acc	Gyr
Accuracy	91	88	95	82	78	80
Precision	95	92	96	83	75	85
Recall	90	87	95	81	77	81
F1	92	89	95	82	76	83

**Table 4:** Testing MSE and MAPE for EE estimation by using two-step progressive training.

Block Combination	AIM-2 Sensor Data						AG Sensor Data					
	Acc+Gyr		Acc		Gyr		Acc+Gyr		Acc		Gyr	
	MSE	MAPE	MSE	MAPE	MSE	MAPE	MSE	MAPE	MSE	MAPE	MSE	MAPE
Block 1 + 2	**0.59**	**0.11**	0.75	0.14	2.03	0.23	1.38	0.15	**0.73**	**0.12**	1.67	0.19
Block 1 + 3	0.66	0.12	0.65	0.13	1.52	0.22	1.02	0.14	0.99	0.15	1.48	0.19

**Table 5:** PAR and EE estimation testing performance for different w_1_ (accelerometer sensor only).

$w_{1}$	AIM-2 Sensor Data					AG Sensor Data				
	PAR			EE Estimation		PAR			EE Estimation	
	Accuracy (%)	Precision (%)	Recall (%)	MSE	MAPE	Accuracy (%)	Precision (%)	Recall (%)	MSE	MAPE
0.1	72	79	72	2.69	0.27	75	71	75	3.02	1.45
0.2	78	78	78	2.71	0.27	79	77	79	2.85	0.31
**0.3**	**80**	**86**	**80**	**2.69**	**0.28**	**80**	**78**	**80**	**2.79**	**0.30**
0.4	80	86	80	2.74	0.28	80	78	80	2.85	0.29
0.5	82	87	82	2.73	0.28	78	78	78	2.89	0.31
0.6	83	87	83	2.85	0.30	78	76	78	2.85	0.29
0.7	84	89	84	2.89	0.30	78	78	78	3.16	0.32
0.8	84	89	84	2.94	0.31	78	76	78	4.46	0.36
0.9	85	90	85	2.83	0.30	78	76	78	2.98	0.29

**Table 6:** PAR testing accuracy for AIM-2 and AG devices using one-step hard parameter sharing training.

Block-1+2, $w_{1}=0.3$ $w_{2}=0.7$	**Average Metric** **(%)**	**AIM-2 Sensor Data**			**AG Sensor Data**		
		**Acc+Gyr**	**Acc**	**Gyr**	**Acc+Gyr**	**Acc**	**Gyr**
	Accuracy	85	80	**88**	76	**80**	69
	Precision	87	86	**91**	74	**78**	73
	Recall	85	80	**88**	76	**80**	69
	F1	86	82	**89**	75	**79**	71

**Table 7:** Testing MSE and MAPE for EE estimation by using one-step hard parameter sharing training.

Block-1+2, $w_{1}=0.3$ $w_{2}=0.7$	**AIM-2 Sensor Data**						**AG Sensor Data**					
	**Acc+Gyr**		**Acc**		**Gyr**		**Acc+Gyr**		**Acc**		**Gyr**	
	**MSE**	**MAPE**	**MSE**	**MAPE**	**MSE**	**MAPE**	**MSE**	**MAPE**	**MSE**	**MAPE**	**MSE**	**MAPE**
	3.90	0.31	**2.69**	**0.28**	3.28	0.28	2.93	0.28	**2.79**	**0.30**	4.30	0.32

**Table 8:** Comparison of studies addressing simultaneous PAR and EE estimations.

Study	Position of Wear	Sensor or Device Used	Number of Activities and Participants	Maximum PAR Performance	Maximum EE Performance	Pros and Cons (Compared to Our Experiment)
[46]	waist	ActiGraph GT9X (acc, gyr, and mag)	7 activities from 10 adults	F1 score of 0.83 (L1-64) and accuracy of 0.98 (L1-64)	MAE of 0.55 (L2-64) METs and MAPE of 17.56% (L2-64)	**Cons:** - EE (MET) calculated from the Friedson adult formula. - Requires an additional gadget for monitoring. **Pros:** - Model validated across various datasets.
[47]	chest and wrist	Cell phone (camera and acc) and heart rate sensors	24 activities from 10 participants	mean average precision of 78.1	Absolute error of 0.696 kcal.m^−1^	**Cons:** - Inconvenience of wearing a cell phone or wearable device on the chest. - EE ground truth is taken from a predefined value. - Privacy concerns of bystanders due to video recording. **Pros:** - Data collected in a free-living environment. - Potential for higher accuracy with the integration of visual and sensor data.
[20]	foot	SmartShoe (force-sensitive resistors and acc sensor)	15 activities from 15 participants	classification accuracy of 95%	Root mean square error (RMSE) of 0.78 kcal/min	**Cons:** - Inconvenience of wearing shoes even during rest (e.g., supine and sitting). - Requires two different models for EE estimation and PAR. **Pros:** - Reduced model complexity, suitable for real-time recognition. - EE ground truth is measured via indirect calorimeter.
[26]	wrist, hip, ankle, upper arm, and thigh	ActiGraph GT3X (acc, gyr, and mag)	32 activities from 93 participants	balanced accuracy for individual activity recognition of 0.42 with the waist sensor	RMSE of 0.91 (hip or thigh position)	**Cons:** - Requires two separate models for EE estimation and PAR. - Involves handcrafted feature selection for model development. - Requires additional gadgets for monitoring. **Pros:** - Includes a larger number of activities and participants. - Compares results across different body positions.
[57]	neck and wrist	Imec’s ECG	6 cluster activities from 16 participants	accuracy of 95%, including the accelerometer and heart rate	RMSE of 1.59 kcal/min with an accelerometer and heart rate	**Cons:** - Discomfort from two gel electrodes on participants’ chests. - Requires additional monitoring gadgets. **Pros:** - Investigate the benefits of three physiological signals, i.e., respiration rate, galvanic skin response, and skin humidity.

## Data Availability

The data are available upon reasonable and responsible request for non-commercial research purposes.
